# Optimal Abdominal CT Image Quality in Non-Lean Patients: Customization of CM Injection Protocols and Low-Energy Acquisitions

**DOI:** 10.3390/diagnostics13132279

**Published:** 2023-07-05

**Authors:** Francesco Macri, Elina Khasanova, Bonnie T. Niu, Anushri Parakh, Manuel Patino, Avinash Kambadakone, Dushyant V. Sahani

**Affiliations:** 1 Department of Radiology, Geneva University Hospitals, University of Geneva, 1211 Geneva, Switzerland; khasanovamd@yahoo.com; 2 Faculty of Medicine, University of Ottawa, Ottawa, ON K1N 6N5, Canada; bonnietn@hotmail.com; 3 Department of Radiology, Abdominal Division, Massachusetts General Hospital, Boston, MA 02114, USA; aparakh1@mgh.harvard.edu (A.P.); patinomanuel1@gmail.com (M.P.); akambadakone@mgh.harvard.edu (A.K.); 4 Department of Radiology, University of Washington, Seattle, WA 98195, USA; dsahani@uw.edu

**Keywords:** computed tomography, contrast material, image quality enhancement, workflow

## Abstract

We compared the image quality of abdominopelvic single-energy CT with 100 kVp (SECT-100 kVp) and dual-energy CT with 65 keV (DECT-65 keV) obtained with customized injection protocols to standard abdominopelvic CT scans (SECT-120 kVp) with fixed volumes of contrast media (CM). We retrospectively included 91 patients (mean age, 60.7 ± 15.8 years) with SECT-100 kVp and 83 (mean age, 60.3 ± 11.7 years) patients with DECT-65 keV in portovenous phase. Total body weight-based customized injection protocols were generated by a software using the following formula: patient weight (kg) × 0.40/contrast concentration (mgI/mL) × 1000. Patients had a prior abdominopelvic SECT-120 kVp with fixed injection. Iopamidol-370 was administered for all examinations. Quantitative and qualitative image quality comparisons were made between customized and fixed injection protocols. Compared to SECT-120 kVp, customized injection yielded a significant reduction in CM volume (mean difference = 9–12 mL; *p* ≤ 0.001) and injection rate (mean differences = 0.2–0.4 mL/s; *p* ≤ 0.001) in all weight categories. Improvements in attenuation, noise, signal-to-noise and contrast-to-noise ratios were observed for both SECT-100 kVp and DECT-65 keV compared to SECT-120 kVp in all weight categories (e.g., pancreas DECT-65 keV, 1.2-attenuation-fold increase vs. SECT-120 kVp; *p* < 0.001). Qualitative scores were ≥4 in 172 cases (98.8.4%) with customized injections and in all cases with fixed injections (100%). These findings suggest that customized CM injection protocols may substantially reduce iodine dose while yielding higher image quality in SECT-100 kVp and DECT-65 keV abdominopelvic scans compared to SECT-120 kVp using fixed CM volumes.

## 1. Introduction

The majority of abdominopelvic CT exams (60–70%) performed in an outpatient setting utilize iodinated intravenous contrast media (CM) [1]. Contrast enhancement is influenced by several factors related to the CM (e.g., iodine dose, injection rate), scanners (e.g., tube voltage, scan delay) and patients (e.g., total body weight, cardiac output) [2].

Total body weight (TBW) has been identified as the most effective factor in the customization of iodine load administered to a patient [2,3,4] due to its strong influence on vascular and solid organ enhancement [3,5]. However, to cope with the constantly growing patient demand to access CT services while maintaining workflow and standardization, a fixed volume of CM is often administered [6,7]. This fixed dosing method can lead to under- or over-CM dosage and yield variable enhancement in patients of different weights [8]. It should be noted that in patients with a BMI > 30 kg/m^2^, there is a possibility of contrast medium (CM) overdosage when adjusting the dose based on total body weight (TBW). However, this issue can be circumvented by adjusting the CM dose based on lean body weight [8].

The customization of CM dose is therefore a desirable practice objective to reduce the variability in interpatient attenuation, possible risk of adverse renal events [9,10] and costs [1] and is now attenable with advances in CT and injector technology. Current CT scanners with powerful tube current generators and iterative reconstructions enable acquisitions with low tube voltage settings (70–100 kVp) in single-energy CT (SECT) and dual-energy CT (DECT). Low-energy acquisitions approach the iodine k-edge (33 keV), increasing iodine attenuation two- to fourfold, which can be harnessed to lessen iodine loads as well as the radiation dose compared to conventional SECT at 120 kVp [11,12,13]. Moreover, DECT allows virtual monochromatic images (VMI; from 40 to 190 keV) to be generated, which are single-photon energy images; in particular, those between 60 and 70 keV provide improved soft tissue image quality compared to the conventional SECT polychromatic images [14]. Other innovations, such as injector software programs (Personalized-patient-protocol-technology (P3T^®^), Certegra^®^; Bayer Healthcare, Berlin, Germany) enable the customization of intravenous contrast volume and injection rate based on TBW. Preliminary results with P3T have proven promising in previous studies, with no difference in image quality for liver imaging when comparing a fixed injection to a customized injection protocol for the same CT scan parameters [15,16,17]. We hypothesized that customizing injection protocols using this injector software program in abdominopelvic CT scans obtained with low-energy SECT and DECT images may result in more appropriate utilization of CM than fixed doses with conventional 120 kVp images.

The purpose of this study is to assess the image quality of low-energy (SECT-100 kVp and DECT-65 keV) abdominopelvic CT images obtained with customized injection protocols compared to conventional SECT-120 kVp ones obtained with fixed CM volume.

## 2. Materials and Methods

### 2.1. Overview

In May 2017, low-energy abdominopelvic CT scans with customized injection protocols had routinely replaced the institutional standard of care CT scan with a fixed CM dosing method in two CT suites in our institution. IRB approval with a waiver for written informed consent was obtained for this HIPPA-compliant, retrospective study.

### 2.2. Patients

**Figure 1:**
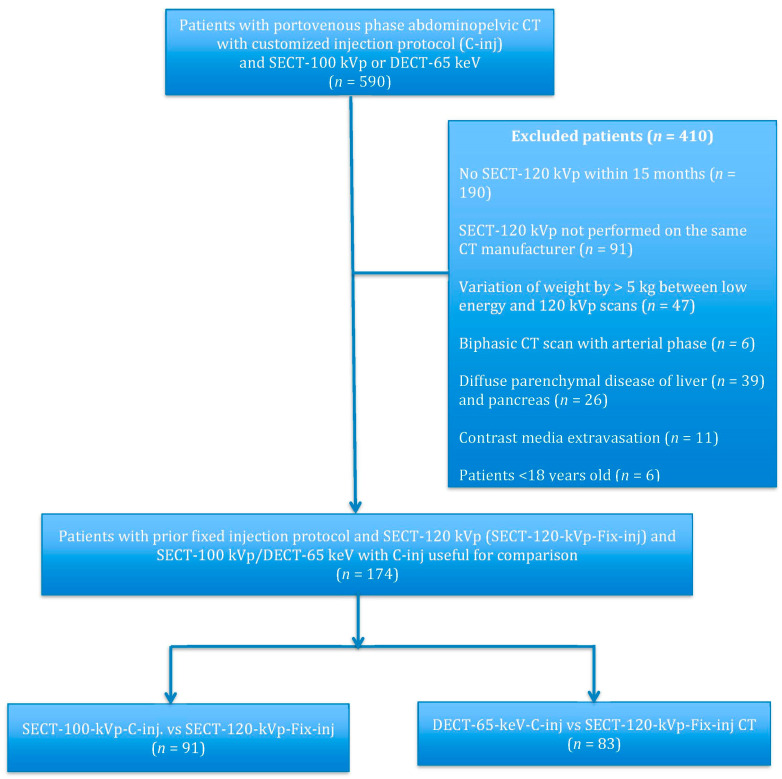
Flowchart of the study population enrollment.

Between 1 September 2017 and 1 April 2018, we retrospectively identified 590 adults who underwent low-energy abdominopelvic CT scans with customized injection protocols in portal venous phase (PVP) for oncological follow-up via picture archiving and communication system (PACS; IMPAX 6.6.1, Agfa HealthCare, Mortsel, Belgium). Patients reported their weight prior to scanning. Patients with the following criteria were excluded: (a) prior standard of care scan performed on a different manufacturer (*n* = 91) to limit inter-manufacturer variations for image quality analysis; (b) lack of prior standard of care CT scan within 15 months (*n* = 190) and more than 5 kg variation in TBW between the two scans (*n* = 47) to decrease intra-patient variability; (c) previous biphasic study with an arterial phase to prevent heterogeneity from different injection protocols parameters (*n* = 6); (d) diffuse parenchymal disease or atrophy (liver: *n* = 39; pancreas: *n* = 26); (e) extravasation of CM (*n* = 11); (f) less than 18 years old (*n* = 6) (Figure 1). Our final cohort consisted of 174 patients (Table 1).

Patients were divided into three weight categories: ≤59.9 kg; 60–89.9 kg; ≥90 kg.

### 2.3. Contrast Media Protocol

Iopamidol (ISOVUE^®^, 370 mgI/mL, Bracco Diagnostics, Monroe Township, NJ, USA) was pre-warmed (37 °C) and administered through a 20–22-gauge cannula (BD Nexiva™, Diffusics™) placed into the antecubital vein using a power injector (Medrad^®^ Stellant^®^, Bayer healthcare, Berlin, Germany) followed by a saline chaser of 40 mL.

The standard CM dosing method for abdominopelvic CT at our institution administers fixed volumes of CM based on the following TBW thresholds: ≤59.9 kg, 80 mL; 60–89.9 kg, 90 mL; ≥90 kg, 120 mL. The injection rate is 2–3 mL/s for monophasic PVP scans.

**Figure 2:**
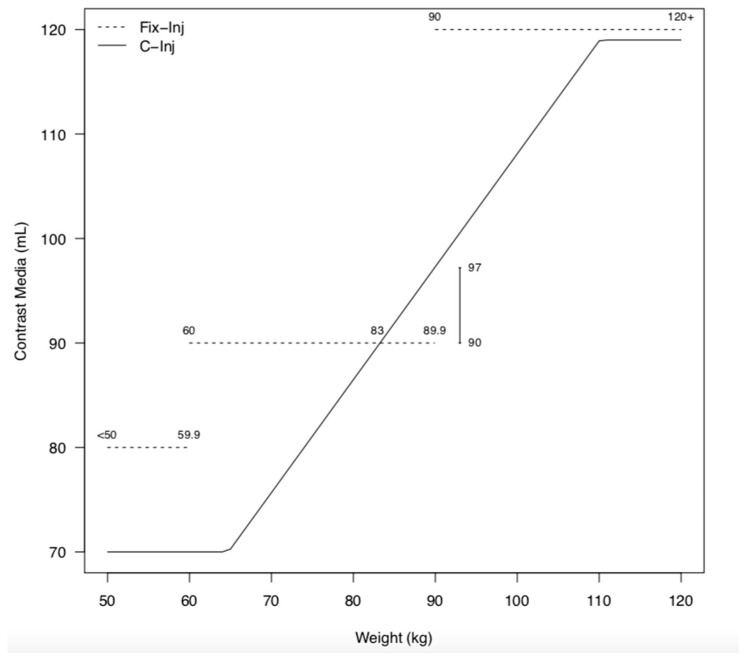
Plot of weight and contrast material. Customized injection (C-inj) resulted in lower contrast media (CM) volume for all weight groups except for individuals who weighed between 83 and 89.9 kg, where an increase by 1–7% mL (91–97 mL) was noted compared to their counterparts with fixed injection (Fix-inj) (90 mL). The reduction in CM volume using the C-inj technique varied by weight group but resulted in markedly reduced volumes (15–23%) for individuals that weighed between 60 and70 kg (70–75 mL vs. 90 mL) and 90 and 95 kg (93–100 mL vs. 120 mL).

P3T abdominal module (Certegra^®^; Bayer Healthcare, Berlin, Germany) was installed in the power injectors and used with default settings in two CT suites. This software utilizes both CM concentration and TBW to personalize the CM volume by using the following formula: patient weight (kg) × weight factor (gI/kg)/contrast concentration (mg/mL) × 1000 [15] (Figure 2). The weight factor was 0.40 gI/kg. For monophasic scans in PVP, the software used fixed injection duration of 40 s and adapted the injection rate accordingly. With these settings, the minimum CM volume delivered by the software in our study cohort was 70 mL.

To compare the results of the customized injections with the fixed injections, patients were divided into the same three weight categories used by the standard CM dosing method.

### 2.4. CT Protocols

All standard of care scans with fixed injections (SECT-120kVp-Fix-inj) were obtained either on a Revolution HD or on a Discovery CT 750HD (GE Healthcare, Milwaukee, WI, USA). Customized injections and low-energy SECT with 100 kVp (SECT-100kVp-C-inj) or DECT reconstructed at a 65 keV monochromatic level (DECT-65keV-C-inj) acquisitions were performed only on the Revolution HD CT scanner. Image acquisition and reconstruction parameters are summarized in Table 2. The scan covered from mid chest to 2 cm below the ischial tuberosities. A bolus tracking ROI was placed in the abdominal aorta, at the level of the celiac trunk, with a trigger at 150 Hounsfield Unit (HU) and acquisition delay at 70 s.

### 2.5. Image Analysis

Quantitative analysis was performed by a radiologist with ten years of experience (R1: blinded for review). Attenuation (HU) and standard deviation (SD) of attenuation were recorded as the average of multiple regions-of-interest (ROIs) within the liver and pancreas. Four measurements (ROI size, 150–200 mm^2^) were obtained from the liver: two ROIs within the right lobe (VIII and VI segments) and two ROIs within the left lobe (II and III segments). Two distinct measurements (ROI size 70–100 mm^2^) were obtained from the pancreatic head and body. Largest possible ROIs were placed while avoiding vessel, ducts, lesions and artifacts.

Image noise was defined as the standard deviation of the ROI of each targeted structure. To assess image quality, we calculated the signal-to-noise ratio (SNR) as follows: mean HU ROI/mean SD ROI. Three ROIs (150–300 mm^2^) were placed in the upper abdominal subcutaneous fat and averaged to calculate the contrast-to-noise ratio (CNR) with the following formula for each structure: ((mean HU structure—mean HU fat)/mean SD fat). All these variables were compared between the low-energy protocols and the standard of care as well as across weight category for each type of protocol. Moreover, since the image quality between CT scans obtained with different tube voltage settings could not be compared independently of the dose, we calculated dose-independent figure of merit (FOM) values as the ratio of CNR^2^ to ED for each structure at each protocol [18].

Qualitative Analysis: Two abdominal radiologists (R1 and R2, blinded for review; R2 with 8 years of experience), who were blinded to scan and contrast protocol, assessed the subjective image quality independently. They assessed the image quality of low-energy CT protocols (SECT-100kVp and DECT-65keV) with customized injections (inter-reader assessment). Each radiologist also evaluated the image quality of low-energy protocols with customized injection compared to that of the standard of care protocol with fixed injections (intra-reader assessment).

Raters viewed the images in an axial plane and were free to modify the preset window settings (width 350, level 50) according to their preference.

Both readers assessed organ contrast enhancement and overall image quality using a 5-point Likert scale (1, unacceptable, very poor organ contrast enhancement with impossibility to distinguish intra organic structures and image noise altering organ margins; 2, poor, suboptimal organ contrast enhancement with difficulty to distinguish intra organic structures and image noise hindering organ margins differentiation; 3, acceptable, somewhat low organ contrast enhancement with distinguishable intra organic structures and image noise not hindering organ margins differentiation; 4, good, substantial organ contrast enhancement with confident definition of intra organic structures and visible organic margins and minimal image noise; 5, excellent, optimal organ contrast enhancement with strikingly visible intra organic structures and organ margins without noticeable image noise). Disagreements were resolved by consensus. The percentage of exact matches for image quality scores ≥ 4 was calculated as the mean value with a corresponding 95% confidence interval.

### 2.6. Radiation Dose Evaluation

The radiation dose information was obtained from the dose report available in PACS. The volumetric CT dose index (CTDI_vol_) and the dose length product (DLP) for the PVP were recorded. Estimates of ED were calculated by multiplying the DLP of each patient with a specific abdomen conversion coefficient k of 0.017 mSv [mGy.cm] [19].

### 2.7. Statistical Analysis

Demographic summaries were calculated for patient cohorts using combinations of imaging techniques (SECT, DECT) and weight groups (≤59.9 kg, 60–89.9, ≥90 kg). Categorical variables were summarized using frequencies and percentages, while continuous variables were summarized using means and standard deviations or medians and interquartile ranges where normalcy and/or equal variance assumptions were not met. Comparisons between imaging technique (SECT or DECT) were performed using either the Chi-square test (χ2; categorical variables) or the analysis of variance (ANOVA; continuous variables). Objective image quality was compared with a paired t-test or the Wilcoxon rank sum test where parametric assumptions were not met. Subjective imaging measures were quantified using absolute agreement percentage with their 95% confidence intervals. A *p*-value of <0.05 was considered statistically significant.

## 3. Results

### 3.1. Patient Population

Of the 174 patients, customized injections with SECT-100 kVp were performed on 91 patients (mean age, 60.3 ± 15.2 years; mean weight, 78.1 ± 20.4 kg; mean BMI, 27.2 ± 6.2 kg/m^2^) and with DECT-65 keV on 83 patients (mean age, 60.0 ± 11.7 years; mean weight, 74.8 ± 18.5 kg; mean BMI, 26.5 ± 4.9 kg/m^2^). No significant intra-patient characteristic differences were observed within the same weight category between the CT scans with customized injection and the prior standard of care CT scans. Patient demographics are presented in Table 1. In SECT-100 kVp group, patients belonging to the lowest weight group were female (mean age, 65.6 years), whereas those belonging to the highest weight group were male (mean age, 55.1 years). Similar characteristics were observed in the DECT-65 keV group.

### 3.2. Contrast Media Injections

Injection rates and contrast media summaries are presented in Table 3 and Figure 2 by imaging technique and weight group.

Injection rates with the customized injection protocols were significantly lower, regardless of the weight group (*p* ≤ 0.037).

CM volume was also significantly lower when using body-weight-customized doses compared to fixed doses (*p* < 0.001; average cohort, 85.3 ± 15.1 mL vs. 94.8 ± 14.8 mL; total iodine load, 410.1 gI/kg vs. 460.1 gI/kg). Although there was an average reduction in CM volume of 9–12 mL, a greater CM volume reduction (15–23%) was observed with patients with customized injections within the weight range of 60–70 kg (70–75 mL vs. 90 mL) and 90–95 kg (93–100 mL vs. 120 mL). Conversely, patients weighing 83–89.9 kg received higher customized amounts of iodine (1–7%; 91–97 mL vs. 90 mL) (Figure 2).

### 3.3. Image Quality

**Figure 3:**
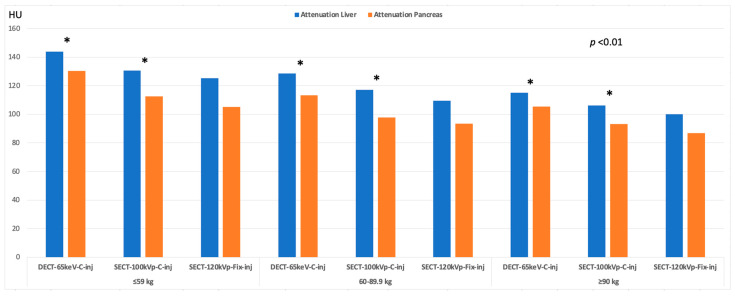
Inter-protocol attenuation (HU) analysis. Bar plot summarizes attenuation values of targeted organs and image technique stratified by weight category. Comparisons were carried out between DECT-65 keV-C-inj and SECT-100-C-inj versus SECT-120-Fix-inj. Asterisk (*) indicates statistically significant difference. Greater attenuation values were observed using customized injection protocols with both DECT-65 keV and SECT-100-kVp in each weight category compared to SECT-120-kVp with fixed injection protocols.

**Figure 4:**
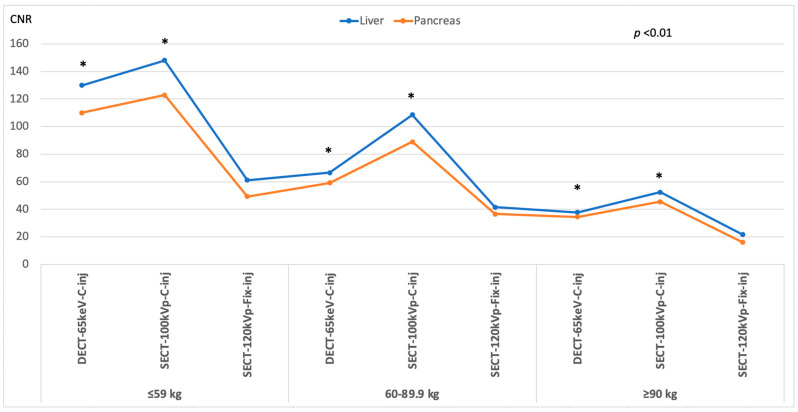
Inter-protocol dose-independent CNR figure of merit (FOM) analysis. Line chart summarizes FOM values of targeted organs and image technique stratified by weight category. Comparisons were carried out between DECT-65 keV-C-inj and SECT-100-C-inj versus SECT-120-Fix-inj. Asterisk (*) indicates statistically significant difference. Higher FOM values were observed using customized injections with both DECT-65 keV and SECT-100-kVp in each weight category compared to SECT-120-kVp with fixed injection protocols.

**Figure 5:**
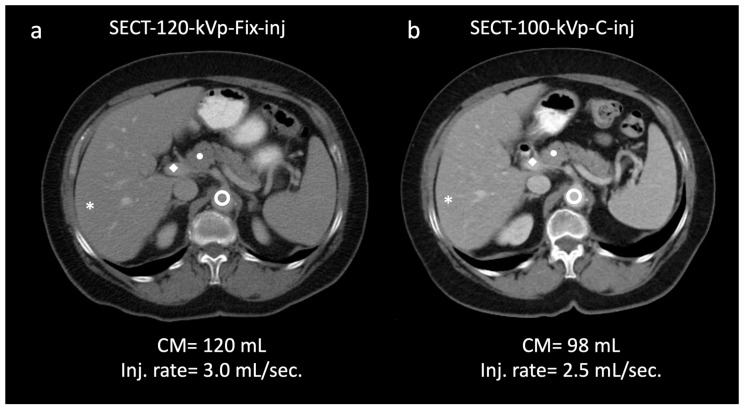
Clinical example of a 62-year-old male (93 kg). A 120 kVp single-energy CT (SECT) with fixed injection (120 mL) demonstrates the following attenuation (HU)/contrast-to-noise ratio (CNR) values: * liver; 97.6/19.6; ● pancreas; 83.8/16.7; 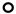 aorta; 145.9/22.8; ◆ portal vein; 157.9/24.7 (**a**). In the same patient, a 100 kVp SECT with customized injection resulted in administration of reduced volume of contrast media and injection rate (98 mL; −15%; 2.5 mL/s; −16.6%, respectively) and yielded significantly higher attenuation/CNR values: * liver; 106.2/26.7 (+8.8/36.2%); ● pancreas; 93.1/22.2 (+11.0/32.9%); 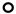 aorta, 167.0/30.6 (+14.4/34.2%); ◆ portal vein, 188.8/32.5 (+19.5/31.5%) (**b**). Subjectively, both readers rated the two scans as 5. Note—Fix-inj, fixed injection; C-inj, customized injection. SECT-100 kVp-C-inj: single-energy CT at 100 kVp with customized injections; SECT-120 kVp-Fix-inj: single-energy CT at 120 kVp with fixed injections.

**Figure 6:**
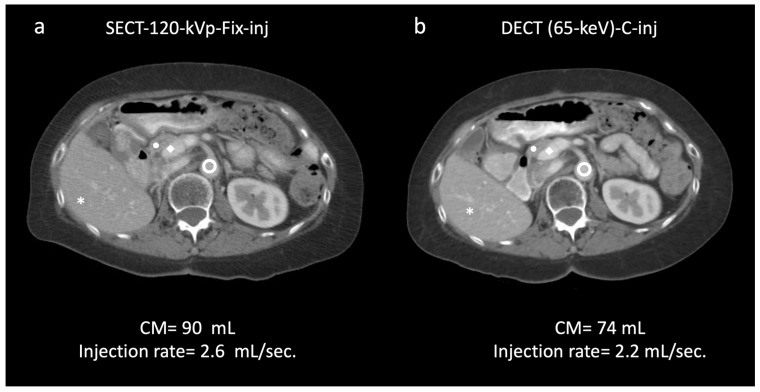
Clinical example of a 58-year-old female (68 kg). A 120 kVp single-energy CT (SECT) with fixed injection (90 mL) demonstrated the following attenuation (HU)/contrast-to-noise ratio (CNR) values: * liver, 114.9/22.5; ● pancreas, 95.4/19.6; 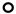 aorta, 149.9/23.5; ◆ portal vein, 162.0/25.6 (**a**). In the same patient, a 65 keV virtual monochromatic image reconstructed from dual-energy CT (DECT) acquisition with customized injection (**b**) resulted in administration of a reduced volume of contrast media and injection rate (74 mL; −17.7%; 2.2 mL/s; −15.3%, respectively) and yielded significantly higher values of attenuation/CNR: * liver, 128.7/30.7 (+12/36.4%); ● pancreas, 113.3/24.6 (+18.7/25.5%); 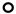 aorta, 186.6/32.7 (+24.4/39.1%); ◆ portal vein, 210.5/32.57 (+29.9/37.1%) (**b**). Subjectively, both readers rated the two scans as 5. DECT-65 keV-C-inj: dual-energy CT at 65 keV with customized injections; SECT-120 kVp-Fix-inj: single-energy CT at 120 kVp with fixed injections.

Results for objective image quality are summarized in Table 4 and Table 5. Attenuation was significantly higher with both SECT-100 kVp and DECT-65 keV protocols with customized injections for all weight categories than with the SECT-120 kVp protocol with fixed injections (*p* ≤0.001; overall average increase: SECT-100 kVp, liver, 7.34% [106.2–130.6 HU]; pancreas, 9.78% [93.1–112.4 HU]; DECT-65 keV, liver, 13.16% [115–143.7 HU]; pancreas, 18.37% [105.4–130.4 HU]) (Figure 3). Image noise was significantly lower in SECT-100 kVp and DECT-65 keV protocols with customized injections than in SECT-120 kVp protocol with fixed injections (*p* ≤0.035). The SNR and CNR for liver and pancreas were significantly greater in images with customized injections compared to those with fixed injections (*p* ≤0.008) (Table 4). CNR dose-independent FOM showed significantly greater values in SECT-100 kVp and DECT-65 keV with customized injections compared to the standard of care (*p* ≤0.039) (Figure 4). Between weight categories (Table 5), no significant attenuation differences were observed between the 60–89.9 kg and ≥90 kg patients (*p* ≥ 0.066) for both organs in SECT-100 kVp and for the pancreas in DECT-65 keV. Conversely, the lightest weight group of patients consistently showed significantly greater attenuation for both organs compared to the two other weight groups in both the SECT-100 kVp and DECT-65 keV scans (*p* ≤ 0.032). Imaging examples are shown in Figure 5 and Figure 6.

All CT scans were considered diagnostic and interpreted prospectively by abdominal imaging radiologists for clinical care. Subjective image quality is presented in Table 6; overall inter-rater absolute agreement was high for at least 98.8% of the cases with customized injections (172/174; rating ≥ 4, 99.4% [95%CI: 96.5–100.0%]) and 100% of the cases with fixed injections (174/174; rating ≥ 4, 100% [95%CI: 97.4–100.0%]). Similar results were obtained across weight groups.

### 3.4. Radiation Dose

Radiation doses (Table 7) were significantly lower in patients imaged with SECT-100-kVp compared to SECT-120-kVp (*p* < 0.001). No significant radiation dose differences were observed between DECT-65 keV and SECT-120-kVp (*p* ≥ 0.078).

## 4. Discussion

Our study demonstrates that the weight-based customization of CM injection protocol is feasible using a software platform for power injectors and yields high-quality images during low-energy abdominopelvic CT exams, achieving an overall reduction in the injection rate and iodine dose.

Conventional CM injection protocols for abdominopelvic CT advocate using 450–600 mg of iodine/kg body weight [20,21]. In our study, the iodine dose per patient ranged from 390–481 mg/kg with a reduction in CM of about 10% on average compared to the standard dosing method.

Very few studies have been conducted on heavy, Western patient cohorts regarding the use of low CT energy levels to reduce iodine load for parenchymal investigations. Martens et al. [17] recently demonstrated that P3T-customized injection protocols at 90 kVp provided good image quality in CT scans with low tube voltage (90 kVp) and decreased total iodine amount versus the standard injection protocol. We delivered a similar total iodine amount (average, 31.8 gI/kg vs. 31.2 gI/kg, respectively) for a cohort with similar mean TBW (76 kg vs. 78 kg), achieving good image quality. When using the same weight stratification as in Martens et al., we administered lower total iodine amount in the 86–90 kg category (−4.5%; 34.4 gI vs. 38.9 gI). Moreover, in our study, lower flow rates were attained, which is of particular interest in an oncology population due to frequently observed poor vein integrity. Conversely to Martens et al. study, we reached attenuation homogeneity across weight categories for the same CT protocol only between patients belonging to the 60–89.9 kg and ≥90 kg categories for both organs in SECT-100 kVp and only for the pancreas for DECT-65 keV (*p*-value ≥ 0.066) (Table 6). The hepatic attenuation in heavy patients, amongst whom there was a higher percentage of steatosis, appeared relatively lower than that of medium-weight patients on DECT-65keV.

Furthermore, patients who weighed ≤ 59.9 kg had significantly higher attenuation for both SECT-100 kVp and DECT-65 keV compared to the other weight categories (*p* ≤ 0.032) (Table 6). This is potentially due to a surplus in the total amount of iodine that, combined with an increased iodine attenuation effect from the low-energy acquisitions, leads to substantially higher attenuating images compared to the medium and large weight categories. Moreover, this effect may have been most pronounced in the thinnest patients of the ≤59.9 kg category who weighed between 40 and 47 kg (*n* = 32/44, 72.2%). Although some differences in noise, SNR and CNR were noted across weight categories within the same CT protocol, the image quality was always excellent.

Clark et al. [22] assessed image quality between defined VMI datasets with reduced iodine amounts compared to standard SECT-120 kVp images with fixed injections in a cohort with a mean TBW similar to ours (76.7 kg vs. 76 kg). They demonstrated that there was a significant increase in attenuation and noise at 52 keV, while no differences were observed at 70 keV versus the standard acquisitions. VMI at 65–70 keV are considered equivalent to 120 kVp with respect to diagnostic image quality and are recommended for CT studies dedicated to parenchymal organs [23]. Compared to Clark and al. [22], our customized injection also allowed for better image quality metrics in DECT-65 keV versus 120 kVp with the additional advantage of lower noise and iodine amount, particularly in larger patients (≥110 kg, 39 gI vs. 46.5 gI).

Other investigators have confirmed the benefits of weight-based CM protocols, resulting in decreased iodine amounts with adequate image contrast in conventional 120 kVp CT exams [16,24,25,26]. Therefore, the conventionally described iodine dosage for abdominopelvic CT scans needs to be revised with the advances in CT technology [27,28] and CM injection methods [15,16,29,30] and the adoption of personalized CT practice in mind. SECT with low tube voltage settings and DECT with low VMI are being increasingly utilized, with subsequent iodine attenuation gain. A drawback of the software is that the default settings generate CM injection protocols based on conventional SECT-120 kVp acquisition parameters. Consistently higher attenuation and SNR and CNR values with acceptable noise image in our comparison groups suggest that we can leverage the use of low energy levels to potentially reduce iodine doses further. Therefore, adjustments to the P3T default software settings might be needed. Another drawback of this software is that the integrity of the intravenous line is not considered on this platform. Hence, the customized injection rate can be inappropriate and may cause extravasation of the CM.

The advantage of using low-energy protocols is two-fold in that it optimizes not only the total iodine amount but also the radiation dose. In our study, SECT-100kVp allowed for significantly lower (*p* < 0.001) and similar radiation doses, respectively, compared to the standard SECT-120 kVp.

An efficient CT workflow is essential to keeping up with high patient volumes. An optimized power injection software can be an adjunct to the technologist for an easier customization of the CM injection protocols, reducing the risk of technologist-related errors and CM waste. The lower CM volume with customized injections also has potential cost-saving benefits in the long term that may be especially more substantial when bulk package bottles are used.

Our study had a few limitations. Firstly, no weight scale was used to measure patients’ weight; patients self-reported their weight to the technologists. Secondly, we did not have control over the injection parameters of SECT-120 kVp performed with fixed injections. The fractional dose was decided by the technologist and varied among patients according to vascular access and clinical task. Thirdly, although all SECT-120 kVp scans were acquired on CT scanners from the same manufacturer, the CT protocol could have slightly varied (different image noise level between low-energy and standard of care CT protocols). Lastly, no assessment of the diagnostic findings was performed. Rather, we focused on the intrinsic image contrast quality, as the CT scans were already deemed diagnostic and reported by abdominal imaging radiologists for clinical care.

## 5. Conclusions

In conclusion, automated customization of injection protocols leads to a decrease in total iodine load while retaining good image quality at low kVp/keV. If the CM software can be adjusted particularly for use of low-energy CT acquisitions, further reductions in CM volume and related cost benefits may be achieved. Moreover, the use of this software could simplify and optimize workflow in busy CT practices.

## Figures and Tables

**Table 1:** Patients’ characteristics.

	≤59.9 kg		60–89.9 kg		≥90 kg	
	SECT-100 kVp	DECT (65 keV)	SECT-100 kVp	DECT (65 keV)	SECT-100 kVp	DECT (65 keV)
N	22	22	41	40	28	21
Male/Female	2/20	2/20	21/20	17/23	22/6	15/6
Age, years	65.6 ± 14.9	62.6 ± 13.3	61.4 ± 15.7	58.1 ± 12.5	55.2 ± 14.1	60.2 ± 6.9
	(22–91)	(37–83)	(24–92)	(27–76)	(25–80)	(45–71)
Body mass index (BMI), kg/m^2^	21.1 ± 2.2	21.8 ± 3.3	26.7 ± 2.2	26.4 ± 3.3	33.2 ± 5.4	31.7 ± 4.1
	(17.1–26.3)	(16.1–32.5)	(21.8–34.1)	(20.0–37.7)	(27.1–53.3)	(27–43.4)
Weight, kg	53.9 ± 3.9	53.0 ± 5.4	74.1 ± 6.2	73.6 ± 8.6	104.4 ± 11.2	99.5 ± 8.3
	(44–59)	(39.5–59)	(60.7–86.1)	(60.3–88.9)	(91–130.6)	(90–114.3)

Data are mean ± standard deviation (range).

**Table 2:** Acquisition and reconstruction parameters.

	CT-1 *	CT-2 **	CT-1/CT-2
Acquisition mode	SECT	DECT	SECT
Tube voltage, kVp (reference) Automatic tube voltage selection (min/max kVp)	100 Present (80/120)	80/140 Not applicable	120 Present (80/120)
Automatic tube current modulation	Enabled For patients ≤ 68.9 kg 200/370 mAs For patients ≥ 69 kg 350/630 mAs		Enabled For patients ≤ 68.9 kg 200/250 mAs For patients ≥ 69 kg 270/330 mAs
Rotation time	0.5		
Collimation	64 × 0.6		
Pitch	0.9		
Iterative reconstruction	ASIR-V 40%	ASIR-V 40%	ASIR-V 40%
Kernel	Abdomen, standard		
Virtual monochromatic level	Not Applicable	65 keV	Not Applicable
Slice thickness	5 mm		
Slice Increment	5 mm		

Note—The automated tube voltage selection yielded 100 kVp scans for all patients in the SECT low tube current group and 120 kVp in patients in the standard of care group. * Revolution HD and ** Discovery CT 750 HD (GE Healthcare, Milwaukee, WI, USA). ASIR: Adaptive statistical iterative reconstruction.

**Table 3:** Injection parameters stratification by weight groups.

Weight	≤59.9 kg			60–89.9 kg			≥90 kg		
SECT-100 kVp-C-inj vs. SECT-120 kVp-Fix-inj									
Injection Protocol	C-inj	Fix-inj	*p*	C-inj	Fix-inj	*p*	C-inj	Fix-inj	*p*
*n*	22			41			28		
Inj. Rate	1.88 ± 0.10	2.26 ± 0.41	0.001	2.31 ± 0.56	2.60 ± 0.47	0.001	2.82 ± 0.43	3.00 ± 0.50	0.037
(mL/s)									
CM volume	70.46 ± 1.71	80.06 ± 0.12	<0.001	81.92 ± 7.12	90.00 ± 0.16	<0.001	109.13 ± 6.21	120.04 ± 0.10	<0.001
(mL)									
**DECT(65keV)-C-inj vs. SECT-120kVp-Fix-inj**									
*n*	22			40			21		
Inj. Rate (mL/s)	2.15 ± 0.67	2.48 ± 0.65	0.001	2.33 ± 0.65	2.74 ± 0.59	<0.001	2.88 ± 0.53	3.07 ± 0.53	0.034
CM volume	70.66 ± 1.20	80.01 ± 0.05	<0.001	80.64 ± 8.26	89.97 ± 0.16	<0.001	107.28 ± 9.06	120.00 ± 0.00	<0.001

Data are mean ± standard deviation. C-inj, customized injections; Fix-inj, fixed injections; Inj. Rate, injection rate; CM, contrast media.

**Table 4:** Inter protocol quantitative image quality.

	≤59.9 kg			60–89.9 kg			≥90 kg		
	C-inj	Fix-inj	*p*-Value	C-inj	Fix-inj	*p*-Value	C-inj	Fix-inj	*p*-Value
SECT-100kVp-C-inj vs. SECT-120kVp-Fix-inj									
Noise									
Liver	10.93	16.52	<0.001	11.47	13.81	<0.001	13.24	16.21	0.013
	±1.86	±4.20		±2.11	±3.06		±3.32	±4.46	
Pancreas	13.99	18.58	<0.001	14.15	17.78	<0.001	16.89	18.29	0.025
	±3.11	±3.67		±3.03	±3.37		±3.27	4.52	
Signal-to-noise ratio									
Liver	12.42	7.96	<0.001	10.57	7.88	<0.001	8.51	6.41	<0.001
	±3.26	±1.65		±2.85	±2.17		±2.54	±2.06	
Pancreas	8.40	5.75	<0.001	7.19	5.36	<0.001	6.13	4.80	<0.001
	±2.13	±1.55		±1.93	±1.45		±1.66	±1.30	
Contrast-to-noise ratio									
Liver	32.61	21.64	<0.001	29.89	22.79	<0.001	26.76	19.65	0.001
	±7.03	±4.32		±6.89	±5.07		±6.01	±6.63	
Pancreas	25.83	18.04	<0.001	24.20	18.43	<0.001	22.22	16.75	<0.001
	±4.47	±3.97		±5.59	±3.74		±4.76	±5.48	
DECT(65-keV)-C-inj vs. SECT-120kVp-Fix-inj									
Noise									
Liver	10.61	14.07	<0.001	12.12	15.06	<0.001	12.81	14.91	0.035
	±2.07	±2.93		±2.67	±2.84		±1.92	±4.11	
Pancreas	13.95	16.99	0.010	15.27	16.55	0.008	16.10	19.39	0.006
	±2.67	±4.67		±2.78	±2.89		±2.38	±4.71	
Signal-to-noise ratio									
Liver	14.12	9.17	<0.001	11.26	7.90	<0.001	9.21	7.41	0.004
	±3.64	±2.04		±3.48	±1.80		±2.11	±2.18	
Pancreas	9.70	6.77	<0.001	7.69	6.05	<0.001	6.63	4.91	<0.001
	±2.29	±1.99		±2.05	±2.22		±1.16	±1.31	
Contrast-to-noise ratio									
Liver	33.57	25.28	<0.001	30.74	22.57	<0.001	22.63	17.63	0.001
	±6.30	±4.86		±7.63	±3.91		±4.73	±3.85	
Pancreas	27.11	20.96	<0.001	22.08	16.47	<0.001	21.70	16.67	0.001
	±4.30	±4.72		±5.72	±3.67		±4.67	±4.14	

Data are mean ± standard deviation. C-inj, customized injections; Fix-inj, fixed injections.

**Table 5:** Intra-protocol quantitative image quality.

	≤59.9 kg	60–89 kg	≥90 kg	Overall *p*-Value	*p*-Value ≤59.9 kg vs. 60–89 kg	*p*-Value ≤59.9 kg vs. 90 kg	*p*-Value 60–89 kg vs. ≥ 90 kg
SECT-100kVp-C-inj							
Attenuation							
Liver	130.60	117.13	106.24	<0.001	0.032	<0.001	NS
	±13.89	±22.55	±20.53				
Pancreas	112.49	97.73	93.131	<0.001	0.002	<0.001	NS
	±15.53	±18.50	±14.63				
Noise							
Liver	10.93	11.47	13.24	<0.01	NS	0.004	0.009
	±1.86	±2.11	±3.32				
Pancreas	13.99	14.15	16.89	0.04	NS	NS	NS
	±3.11	±3.03	±3.27				
Signal-to-noise ratio							
Liver	12.42	10.57	8.51	<0.001	0.037	<0.001	0.008
	±3.26	±2.85	±2.54				
Pancreas	8.40	7.19	6.13	<0.001	0.042	<0.001	NS
	±2.13	±1.93	±1.66				
Contrast-to-noise ratio							
Liver	32.61	29.89	26.76	NS	NS	NS	NS
	±7.03	±6.89	±6.01				
Pancreas	25.83	24.20	22.22	NS	NS	NS	NS
	±4.47	±5.59	±4.76				
DECT(65-keV)-C-inj							
Attenuation							
Liver	143.77	128.71	115.04	<0.001	<0.001	<0.001	0.002
	±17.24	±12.17	±15.76				
Pancreas	130.46	113.33	105.48	<0.001	<0.001	<0.001	NS
	±16.73	±16.91	±18.49				
Noise							
Liver	10.61	12.12	12.81	<0.010	0.046	0.008	NS
	±2.07	±2.67	±1.92				
Pancreas	13.95	15.27	16.10	0.031	NS	0.025	NS
	±2.67	±2.78	±2.38				
Signal-to-noise ratio							
Liver	14.12	11.26	9.21	<0.001	0.003	<0.001	NS
	±3.64	±3.48	±2.11				
Pancreas	9.70	7.69	6.63	<0.001	<0.001	<0.001	NS
	±2.29	±2.05	±1.16				
Contrast-to-noise ratio							
Liver	33.57	30.74	22.63	<0.001	<0.001	<0.001	NS
	±6.30	±7.63	±4.73				
Pancreas	27.11	22.08	21.70	<0.001	0.001	0.002	NS
	±4.30	±5.72	±4.67				
SECT-120kVp-Fix-inj							
Attenuation							
Liver	115.27	101.51	93.41	<0.001	<0.001	<0.001	0.012
	±18.66	±19.04	±18.86				
Pancreas	105.25	93.53	86.75	<0.001	<0.001	<0.001	0.040
	±16.23	±15.23	±15.77				
Noise							
Liver	16.54	15.80	17.21	NS	NS	NS	NS
	±4.18	±3.40	±4.70				
Pancreas	17.78	17.20	18.75	NS	NS	NS	NS
	±4.23	±3.20	±4.58				
Signal-to-noise ratio							
Liver	7.41	6.79	5.84	<0.001	NS	<0.001	0.010
	±2.20	±2.22	±2.01				
Pancreas	6.26	5.69	4.85	<0.001	0.046	<0.001	0.013
	±1.84	±1.87	±1.28				
Contrast-to-noise ratio							
Liver	19.93	17.11	16.23	<0.001	<0.001	<0.001	NS
	±5.97	±4.45	±5.24				
Pancreas	18.40	16.45	15.94	0.033	0.076	0.037	NS
	±5.33	±4.23	±5.24				

Data are mean ± standard deviation. Comparisons were carried out across weight categories within the same CT protocol. NS, not significant.

**Table 6:** Subjective quality depicting inter-rater and intra-rater assessment for image quality.

	SECT-100 kVp	DECT-65 keV
R1 vs. R2	100 (95.3–100.0)	98.8 (92.5–99.9)
Customized injections	n = 174/174	n = 172/174
R1 customized vs. fixed injections	100 (95.3–100.0)	98.8 (92.5–99.9)
	n = 174/174	n = 172/174
R2 customized vs. fixed injections	100 (95.3–100.0)	98.8 (92.5–99.9)
	n = 174/174	n = 172/174

Note Fix-inj; fixed injection; C-inj; customized injection. Data are mean percentages (95% confidence interval). Percentages describe an inter- and intra-reader exact match for image quality score ≥ 4. R1, reader 1; R2, reader 2.

**Table 7:** Radiation dose results stratified by weight and CT technique.

Radiation Dose									
	≤59.9 kg			60–89.9 kg			≥90 kg		
	SECT-100-kVp-C-inj	SECT-120-kVp-Fix-inj	*p*-Value	SECT-100-kVp-C-inj	SECT-120-kVp-Fix-inj	*p*-Value	SECT-100-kVp-C-inj	SECT-120-kVp-Fix-inj	*p*-Value
Number of patients	22			41			28		
CTDI_vol_, mGy	5.10 ±0.91	7.61 ±2.37	<0.001	6.61 ±1.84	10.93 ±2.63	<0.001	10.47 ±2.72	15.83 ±4.26	<0.001
DLP, mGy*cm	245.29 ±41.63	368.77 ±115.44		325.58 ±84.64	531.77 ±126.94		573.78 ±159.81	830.66 ±253.92	
ED, mSv	4.16 ±0.70	6.26 ±1.96		5.53 ±1.43	9.04 ±2.15		9.75 ±2.71	14.13 ±4.31	
	**DECT-C-inj**	**SECT-120-kVp-Fix-inj**		**DECT-C-inj**	**SECT-120-kVp-Fix-inj**		**DECT-C-inj**	**SECT-120-kVp-Fix-inj**	
Number of patients	22			40			21		
CTDI_vol_, mGy	8.29 ±2.41	7.47 ±2.40	0.182	10.69 ±3.58	11.32 ±2.40	0.179	17.19 ±3.44	17.05 ±1.77	0.877
DLP, mGy*cm	392.72 ±117.20	335.52 ±104.97	0.078	552.14 ±192.13	562.07 ±121.12	0.715	877.20 ±207.30	842.24 ±163.88	0.623
ED, mSv	6.67 ±1.99	5.70 ±1.78		9.40 ±3.22	9.60 ±2.05		14.91 ±3.52	14.31 ±2.78	

Data are mean ± standard deviation. CTDIvol, volumetric CT dose index; DLP, dose length product; ED, effective dose; C-inj, customized injections; Fix-inj, fixed injections.

## Data Availability

Not applicable.
